# Anaesthesia in patients undergoing cytoreductive surgery with hyperthermic intraperitoneal chemotherapy: retrospective analysis of a single centre three-year experience

**DOI:** 10.1186/1477-7819-12-136

**Published:** 2014-05-01

**Authors:** Marie-Elisabeth Kajdi, Beatrice Beck-Schimmer, Ulrike Held, Reto Kofmehl, Kuno Lehmann, Michael Thomas Ganter

**Affiliations:** 1 Institute of Anaesthesiology, University Hospital Zurich, Raemistrasse 100, 8006 Zurich, Switzerland; 2 Horten Centre for Patient Oriented Research and Knowledge Transfer, University Hospital Zurich, Pestalozzistrasse 24, 8091 Zurich, Switzerland; 3 Department of Surgery, University Hospital Zurich, Raemistrasse 100, 8006 Zurich, Switzerland; 4 Institute of Anaesthesiology and Pain Medicine, Kantonsspital Winterthur, Brauerstrasse 15, Postfach 834, 8401 Winterthur, Switzerland

**Keywords:** Anaesthesia, General, Chemotherapy, Cancer, Regional perfusion, Fluid therapy, Hyperthermia, Induced, Perioperative care

## Abstract

**Background:**

Cytoreductive surgery combined with hyperthermic intraperitoneal chemotherapy (CRS/HIPEC) is a treatment option for selected patients with peritoneal carcinomatosis. There are limited data available on anaesthesia management and its impact on patients’ outcome. Our aim was to retrospectively analyze and evaluate perioperative management and the clinical course of patients undergoing CRS/HIPEC within a three-year period.

**Methods:**

After ethic committee approval, patient charts were retrospectively reviewed for patient characteristics, interventions, perioperative management, postoperative course, and complications. Analysis was intervention based. Data are presented as median (range).

**Results:**

Between 2009 and 2011, 54 consecutive patients underwent 57 interventions; median anaesthesia time was 715 (range 370 to 1135) minutes. HIPEC induced hyperthermia with an overall median peak temperature of 38.1 (35.7-40.2)°C with active cooling. Bleeding, expressed as median blood loss was 0.8 (0 to 6) litre and large fluid shifts occurred, requiring a total fluid input of 8.4 (4.2 to 29.4) litres per patient. Postoperative renal function was dependent on preoperative function and the type of fluids used. Administration of hydroxyethyl starch colloid solution had a significant negative impact on renal function, especially in younger patients. Major complications occurred after 12 procedures leading to death in 2 patients. Procedure time and need for blood transfusion were associated with a significantly higher risk for major complications.

**Conclusions:**

Cytoreductive surgery with HIPEC is a high-risk surgical procedure associated with major hemodynamic and metabolic changes. As well as primary disease and complexity of surgery, we have shown that anaesthesia management, the type and amount of fluids used, and blood transfusions may also have a significant effect on patients’ outcome.

## Background

Over the last two decades, cytoreductive surgery combined with hyperthermic intraoperative chemotherapy (CRS/HIPEC) has become a therapeutic option for selected patients with peritoneal carcinomatosis [1]. Traditionally, peritoneal carcinomatosis was considered a palliative incurable condition [2]. Sugarbaker [3], however, first described that some of these patients may benefit from the surgical removal of all macroscopic tumor, combined with locoregional chemotherapy [3]. Since then, CRS/HIPEC has increasingly been used to treat patients with peritoneal carcinomatosis of different origin [4-11].

Strict patient selection is crucial and meticulous surgical tumor removal is mandatory for the best clinical outcome [9,12-14]. Thereby, longterm survival with good quality of life is feasible [15]. As there is a learning curve when performing CRS/HIPEC, centralization of the procedure to specialized institutions is recommended [16]. Regarding anaesthesia management and perioperative care, experience is limited and a consensus has yet to be found [17]. Several authors have shown major changes in body temperature and hemodynamics, alterations in the composition of the blood as well as need for massive transfusion [18-21].

The aim of our study was to retrospectively analyze anaesthesia management and postoperative course of patients undergoing CRS/HIPEC over a 3-year period since introduction of this combined technique at the University Hospital Zurich.

## Methods

After ethic committee approval (Kantonale Ethik Kommission, 8090 Zurich, Switzerland; KEK# 2012–0174), all patients operated on in a three-year period between 2009 and 2011 were included from a prospective database. Charts were retrospectively reviewed. There were no exclusion criteria. A total of 54 patients underwent 57 procedures in the time frame specified. Data analysis was based on the number of procedures (57 = 100%).

### Data collection and study variables

**Figure 1:**
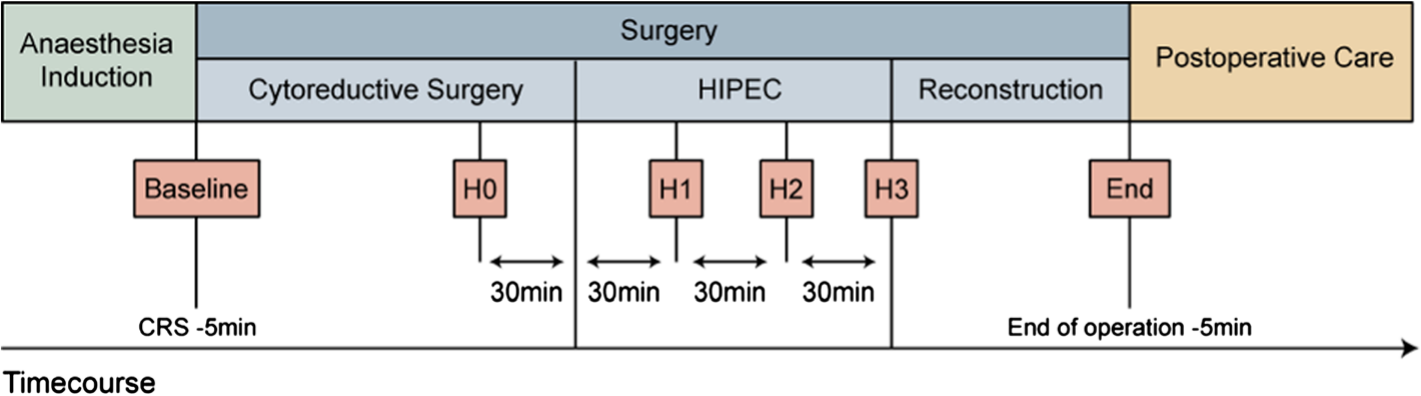
**Time course of procedure.** baseline = after induction of anaesthesia but 5 minutes before start of the operation, H0 = 30 minutes before HIPEC, H1 and H2 = 30 and 60 minutes after start of HIPEC, H3 = end of HIPEC, End = 5 minutes before end of the operation. CRS, cytoreductive surgery; HIPEC, hyperthermic intraperitoneal chemotherapy.

Anaesthesia and perioperative data were collected from electronic patient records (KISIM™, CISTEC AG, Zurich, Switzerland). Surgery was divided into three phases: CRS, HIPEC, and reconstruction. Furthermore, we defined six particular time points in order to describe the course of the intervention (Figure 1). Data were collected on patient characteristics, anaesthesia, intraoperative fluid, transfusion and coagulation management, microcirculation, and body temperature. Laboratory values and blood gas analysis were recorded until the second postoperative day. Additionally, the postoperative course including complications according to the Clavien-Dindo classification were recorded. Major complications included re-interventions under general anaesthesia (grade 3b), life-threatening complications requiring ICU management (grade 4), and death (grade 5) [22].

### Cytoreductive surgery and HIPEC

All patients underwent extensive CRS followed by HIPEC. A peritonectomy was performed as described by Sugarbaker [3]. For HIPEC, the open abdomen technique (also referred to as the ‘coliseum technique’) was used, allowing the surgeons to manipulate abdominal content [23]. Inflow and outflow tubes were connected to the hyperthermia pump (Belmont™ Hyperthermia Pump, Belmont Instrument Corporation, Billerica, United States) and 750 to 1000 ml min^−1^ of preheated 1.5% glucose peritoneal dialysis solution was circulated through the abdominal cavity. When the target temperature of between 41 and 42°C was reached, chemotherapeutic agents were added to the solution. Three different chemotherapeutic regimens were used: doxorubicin combined with mitomycin, doxorubicin combined with cisplatin, and cisplatin combined with mitomycin. HIPEC was scheduled for 60 or 90 minutes; afterwards, the perfusate was drained and the abdominal cavity washed out with 4000 ml of normal saline (37°C). To prevent systemic hyperthermia, active cooling with forced air, cold packs, and an infusion of cold fluids (4°C) was used.

### Anaesthesia and postoperative care

Anaesthesia was performed according to institutional guidelines, with propofol or volatile anaesthetics with restrictive transfusion management, and extensive hemodynamic monitoring. Combined anaesthesia, including continuous thoracic epidural anaesthesia (TEA), was the technique of choice. After surgery, patients were transferred to the ICU or post-anaesthesia care unit (PACU). However, due to the lack of standardization at this early stage, individual management was up to the anesthesiologist in care.

### Guidance of hemodynamic and fluid therapy

To maintain end-organ perfusion, general fluid management included a continuous baseline infusion of crystalloids, aiming at a urinary output of at least 0.5 (CRS phase), 2 (HIPEC phase), and 1 (reconstruction phase) ml kg^−1^ h^−1^, respectively. If necessary, norepinephrine was continuously applied to keep mean arterial blood pressure at ± 20% of baseline values. Arterial blood gas analyses were drawn to monitor signs of tissue hypoperfusion such as decreasing pH and base excess or increasing serum lactate levels. Volume trials were initiated if defined urinary output was not achieved and/or signs of impaired microcirculation were present. If applicable, the PiCCO system [Pulsion Medical Systems, Munich, Germany] was used for goal-directed hemodynamic and fluid management. In a steady state, with surgical manipulation absent, the parameters of transpulmonary thermodilution and pulse contour analysis were acquired: stroke volume variation (SVV) of higher than 10% was considered a marker for volume responsiveness. Changes in cardiac output, global end-diastolic volume, and extravascular lung water indexes were monitored and used as markers for further fluid trials or vasopressors according to the manufacturer’s hemodynamic decision model [24].

### Statistical methods

Data were extracted from patient records and stored in an Excel file (Microsoft Office 2011). Descriptive statistics are presented as median and ranges for continuous variables and as counts for categorical variables. Intraoperative changes of body temperature, heart rate, mean arterial blood pressure (MAP), and central venous pressure (CVP) were addressed separately with mixed-effect models, accounting for repeated observations over time, and adjusting for the potential confounders of age, gender, and body mass index (BMI). The Box-Cox transformed glomerular filtration rate (GFR) measured postoperatively (day one and two) was modelled with multiple linear regression. Independent factors were preoperative GFR, blood loss, urine output, and different intravenous fluid preparations (Additional file 1: Table S1). For binary outcomes such as postoperative ventilation and major (≥3b) complications, multiple logistic regression models were used. Independent factors are shown in Additional file 2: Table S2. Resulting effect sizes correspond to the logarithm of the odds ratios (OR).

The fentanyl consumption per kg body mass, length of postoperative ventilation, time to first bowel passage, and the length of stay on the ICU between the groups of patients with and without additional thoracic epidural anaesthesia (TEA) were compared with a non-parametric Wilcoxon test (Additional file 3: Table S3). *P* values of <0.05 were considered statistically significant.

All statistical procedures ignored the fact that three patients had two HIPEC interventions, and observations were considered independent. All analyses were performed with R, a software environment for statistical computing and graphics [25]. The R package “reporttools” was used for obtaining descriptive statistics, and the R package “lme4” was used for fitting the random effects models. Details of statistical analyses are presented as supplementary data (Additional file 1: Table S1, Additional file 2: Table S2 and Additional file 3: Table S3).

## Results

Data on patient characteristics and primary cancer are presented in Table 1. A total of 54 patients underwent 57 interventions. The median BMI was 25 (range 16 to 41). The majority of patients suffered from cancer originating from the vermiform appendix. Other primary tumor localizations included colorectal and gastric cancer, mesothelioma, endometrioid and ovarian cancer, and cancer arising from the urachus and small intestine. The median operation time was 550 (255 to 995) minutes and the median anaesthesia time was 715 (370 to 1135) minutes. The median time was 340 (95 to 790) minutes for CRS, 90 (60–115) minutes for HIPEC, and 119 (40 to 237) minutes for reconstruction.

**Table 1:** Patient characteristics, primary cancer, and intraperitoneal chemotherapy

**Age**; years	52 (20–72)
**Gender**; Male:Female	23:34
**Weight;** kg / **Height;** cm	70 (44–112) / 168 (155–185)
**BMI**; kg m^−2^	25 (16–41)
**ASA class** I/II/III	5/49/3
**Comorbidities**^1^	
Cardiovascular	14
Pulmonary	5
Renal	4
Endocrine	3
Neurological	4
Obesity (BMI >30)	8
**Medication**	
Single ß-blocking agent	2
Single ACE-inhibitor/AT2-blocker	3
Single diuretic	0
Combination of at least two drugs	6
Other drugs^2^	27
None	23
**Origin of primary cancer**	
Appendix	33
Ovary	1
Colorectal	13
Mesothelioma	5
Gastric	1
Other origin^3^	4

54 patients underwent 57 procedures. Data are expressed as median (range) and numbers.

ACE, angiotensin converting enzyme; ASA, American Society of Anesthesiology; AT2, angiotensin II; BMI, body mass index (kg m^−2^).

^1^Comorbidities: *cardiovascular* (arterial hypertension, cardiac valve pathology, hyperlipidemia); *pulmonary* (chronic bronchitis, asthma, obstructive sleep apnea syndrome, history of acute respiratory distress syndrome, history of pulmonary embolism); *renal* (one sided kidney agenesia, history of carcinoma of the kidney, incidentaloma, chronic kidney disease); *endocrine* (previous ovariectomy, medically treated hypothyroidism, diabetes mellitus); *neurological* (polyneuropathy, paraesthesia, migraine, herniated vertebral disc with neurological symptoms).

^2^Twenty seven patients were on additional medication: analgesics (n = 9), proton pump inhibitors (n = 6), vitamins and supplements (n = 5), laxatives (n = 5), hormone replacement therapy (n = 5), sedatives (n = 3), oral antidiabetics (n = 1), chemotherapeutic agents (n = 1), antidepressants (n = 1), antidiarrheals (n = 1), antiemetics (n = 1), acetylsalicylic acid (n = 1), statins (n = 1), calcium channel blocker (n = 1), herbal and homeopathic preparations (n = 2).

^3^Other origin summarizes endometrioid cancer (n = 2), cancer of the small intestine (n = 1) and urachus cancer (n = 1).

### Anaesthesia and monitoring

Data on intra- and peri-operative parameters are presented in Table 2. In addition to routine monitoring, advanced hemodynamic monitoring was used in 91% of all procedures (PiCCO (Pulsion Medical Systems, Munich, Germany) n = 48; pulmonary artery catheter (Swan-Ganz CCOmbo, Edwards Life Sciences, Unterschleissheim, Germany) n = 3; both techniques n = 1). General anaesthesia was performed according to institutional standards, with 79% (n = 45) being combined with a continuous thoracic epidural anaesthesia (TEA, ropivacaine 0.33% at between 6 and 12 ml h^−1^). Anaesthesia was maintained with propofol (n = 37), sevoflurane (n = 17), or desflurane (n = 3) and supplemented with intravenous fentanyl according to patients’ needs. Overall median fentanyl consumption was 1.2 (range between 0.2 and 4.0) mg. Thoracic epidural anaesthesia was maintained postoperatively with ropivacaine 0.2% at a rate of between 6 and 15 ml h^−1^.

**Table 2:** Intra- and peri-operative parameters

**Anaesthesia time;** minutes	715 (370–1135)
**Additional thoracic epidural anaesthesia**	45
**Advanced hemodynamic monitoring**	
PiCCO	48
Pulmonary artery catheter	3
Both	1
**Anaesthesia maintenance**	
Propofol	37
Sevoflurane	17
Desflurane	3
**Cumulative fentanyl dose**; mg	1.2 (0.2-4.0)
**Effective operation time**; minutes	550 (255–995)
Length of CRS; minutes	340 (95–790)
Length of HIPEC, minutes	90 (60–115)
Length of reconstruction; minutes	119 (40–237)
**Intraperitoneal chemotherapy** (mg m^−2^)	
Doxorubicin and mitomycin (15 and 15)	49
Doxorubicin and cisplatin (15 and 50)	6
Cisplatin and mitomycin (17 and 10)^1^	2
**Transfer to ICU**	53
**Length of ICU stay**; days	2 (1–35)
**Postoperative ventilation**	33
**Length of postoperative ventilation**; hours^2^	4 (1–10)
**Hospital stay**; days	17 (9–259)

Data presented as median (range) or numbers (n).

CRS, Cytoreductive surgery; HIPEC, hyperthermic intraperitoneal chemotherapy.

Hemodynamic monitoring: PiCCO (Pulsion Medical Systems, Munich, Germany); Pulmonary artery catheter (Swan-Ganz CCOmbo, Edwards Life Sciences, Unterschleissheim, Germany).

^1^Reduced dose of cisplatin was given in n = 2 patients.

^2^Data missing in n = 6 patients.

### Body temperature and hemodynamics

**Figure 2:**
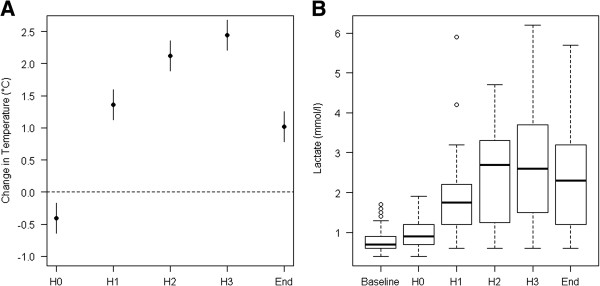
**Intraoperative course of temperature and lactate. A**. Change in temperature compared to baseline: the horizontal line set at 0 is representing baseline. If the 95% confidence interval presented for each time point does not overlap with baseline, temperature differs significantly from baseline (*P* <0.05). A mixed-effect model describing the effect of phase was used. **B**. Boxplot describing arterial lactate levels throughout the intervention. Baseline = after induction of anaesthesia but before start of the operation, H0 = 30 minutes before HIPEC, H1 and H2 = 30 and 60 minutes after start of HIPEC, H3 = end of HIPEC, End = 5 minutes before end of the operation. HIPEC, hyperthermic intraperitoneal chemotherapy.

HIPEC induced hyperthermia with a median overall peak temperature of 38.1 (35.7 to 40.2)°C. Body temperature changed significantly over time (Figure 2A).

The following hemodynamic changes were found (data not shown): heart rate significantly increased throughout the procedure, peaked at the end of HIPEC, and remained high until the end of surgery. Mean arterial blood pressure was kept within 10% of baseline. Norepinephrine was administered in 55 patients, with a median overall peak dose of 7 (0.5 to 30) μg min^−1^. The median central venous pressure (CVP) increased significantly during the first part of the operation (H0 to H2).

### Fluid and coagulation management

Detailed information on intraoperative fluid, transfusion, and coagulation management is shown in Table 3. Coagulation parameters were analyzed using routine laboratory testing and bedside rotational thromboelastometry (ROTEM™, Tem Innovations GmbH, Munich, Germany). One patient with a bleeding diathesis suffering from a known hereditary factor VII deficiency required recombinant factor VIIa. Postoperatively, seven patients showed thrombocytopenia (<50000 μl^−1^) and nine patients developed leukocytopenia (<4000 μl^−1^) on median postoperative day three (range 0 to 12). Preoperative median hemoglobin values were 127 (range 97 to 164) g l^−1^, falling to 82 (range 46 to 125) g l^−1^ intraoperatively. At the end of surgery, the median hemoglobin level was 92 (range 59 to 128) g l^−1^ and remained low until postoperative day two.

**Table 3:** Perioperative fluid balance, blood loss, and substitution

	**n**	**Median (range)**
**Input**		
***Fluids***		
Crystalloids; ml	57	5900 (2200–19100)
*Crystalloids per hour;* ml h^−1^		473 (187–1041)
Colloids; ml	56	2500 (500–14500)
*Colloids per hour;* ml h^−1^		189 (52–852)
HES 130/0.4; ml	14	1000 (500–2500)
Gelatine; ml	51	2500 (500–12000)
***Blood products and coagulation factor concentrates***		
PRBC; n	16	4 (1–10)
FFP; n	3	6 (4–8)
Thrombocytes; n	4	1 (1–2)
Fibrinogen; g	21	4 (2–22)
Prothrombin complex concentrate; IU	9	1000 (400–2000)
Factor XIII; IU	13	1500 (1250–4000)
Factor VIII-vWF; IU	1	1000
Recombinant factor VII; μg	1	1000
**Total input; ml**	57	8200 (4200–29400)
***Total hourly input;*** **ml h**^**−1**^		697 (363–1603)
**Output**		
Blood loss; ml	57	800 (0–6000)
Urine; ml	57	1460 (330–3970)
*Urine per hour,* CRS; ml h^−1^		94 (34–350)
*Urine per hour,* HIPEC; ml h^−1^		220 (47–787)
*Urine per hour,* reconstruction; ml h^−1^		183 (33–631)
Ascites; ml	11*	1500 (100–3000)
**Total output; ml**	57	2670 (530–10780)
***Total hourly output;*** **ml h**^**−1**^		218 (58–729)

54 patients underwent 57 procedures. Data are expressed as median (range) and numbers (n).

Fluids: Crystalloids = Ringerfundin™ (B.Braun Medical AG, Melsungen, Germany); Colloids = gelatine (Physiogel™ balanced, B. Braun Medical AG, Melsungen, Germany) and HES 130/0.4 (Tetraspan™, B. Braun Medical AG, Melsungen, Germany). PRBC and thrombocytes were applied in units of 300 ml, FFP in units of 280 ml.

Coagulation factor concentrates: Fibrinogen (Hemocomplettan P™, CSL Behring AG, Bern, Switzerland), Prothrombin complex concentrate (PCC, Beriplex P/N™, CSL Behring AG, Bern, Switzerland), Factor XIII (Fibrogammin P™, CSL Behring AG, Bern, Switzerland), Factor VIII-vWF (Hemate P™, CSL Behring AG, Bern, Switzerland), recombinant activated Factor VIIa (Novoseven™, Novo Nordisk Pharma AG, Kusnacht, Switzerland).

*data missing in 46 patients.

HES, hydroxyethyl starch; IU, international units; PRBC, packed red blood cells; FFP, fresh frozen plasma; vWF, von Willebrand factor.

### Renal function and metabolic alterations

Details on urine output are summarized in Table 3. To maintain urine output during HIPEC, fluids in combination with IV diuretics were administered to 35 patients (61%). Furosemide was administered to 25 patients (44%), mannitol was administered 20 patients (35%) and 10 patients (18%) received both drugs. Median doses were 10 (range 2.5 to 20) mg for furosemide and 20 (range 20 to 40) g for mannitol. Two patients became oliguric (urine output <0.5 ml kg^−1^ h^−1^) without clinical relevance. Preoperative GFR had an impact on postoperative GFR as the higher the preoperative value, the higher the postoperative value (*P* <0.001) (Additional file 1: Table S1). Intraoperative blood loss and urine output had no significant impact on postoperative GFR (Additional file 1: Table S1). Regarding the type of fluid administered, we did not find any negative effects of crystalloids on renal function. However, the amount of hydroxyl-ethyl starch (HES) given had a significant negative effect on postoperative GFR in patients younger than 60 years (*P* <0.001) (Additional file 1: Table S1). Three patients (5%) suffered from acute deterioration of renal function during their hospital stay.

During surgery, pH and base excess decreased significantly. The lowest values were reached at the end of HIPEC (stage H3) with a median pH of 7.38 (range 7.27 to 7.53) and a median base excess of −4.3 (range −10.8 to 0.6) mEql l^−1^. Figure 2B describes plasma lactate levels inversely increasing throughout the intervention. Hyperglycemia, defined as a blood glucose level of >10 mmol l^−1^, was present in 42 patients (74%). Sixteen patients (28%) required insulin therapy intraoperatively, although only one of the patients was a known diabetic.

### Postoperative course

**Figure 3:**
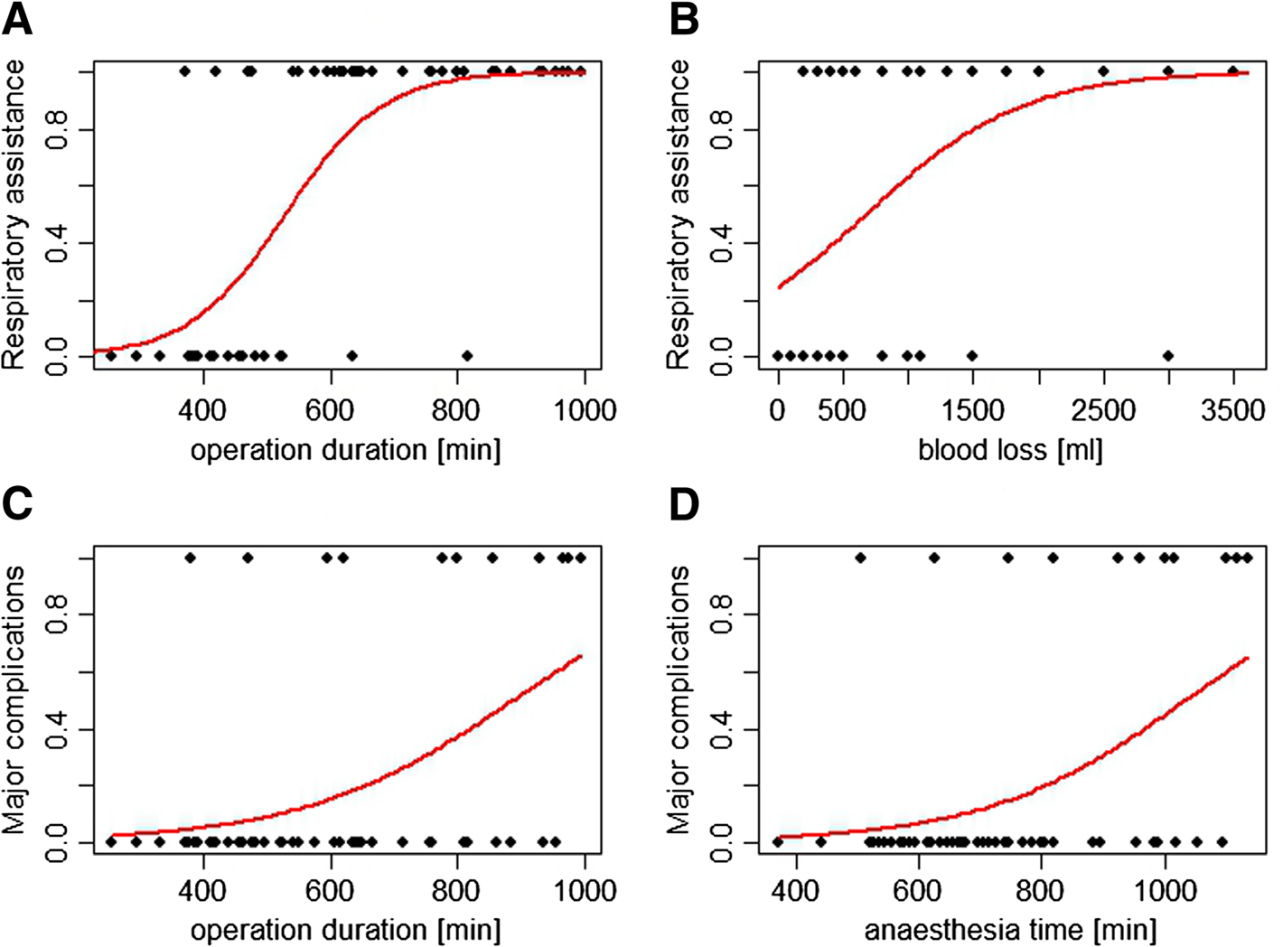
**Operation time, blood loss, and anaesthesia time and their effects on the need for postoperative ventilation and major surgical complications. A** and **B**. The multiple logistic regression model describes the need for postoperative respiratory assistance (vertical axis: 0 = no assistance, 1 = assistance needed) depending on operation time (minutes) and blood loss (ml). The longer the operation (*P* <0.01) and the higher the blood loss (*P* <0.05), the higher the need was for postoperative ventilation. **C** and **D**. Major complications (≥3b according to the Clavien-Dindo classification) on the vertical axis (0 for complications <3b, 1 for ≥3b) are plotted against operation time (minutes) or anaesthesia time (minutes) on the horizontal axis. The longer the operation (*P* <0.01) and the longer anaesthesia time (*P* <0.01), the higher the incidence of major complication was. Data are corrected for BMI and age.

The median length of hospital stay was 17 (range 9 to 259) days and the median length of ICU stay was 2 (range 1 to 35) days. A total of 33 ICU patients were ventilated on ICU arrival. There was a positive correlation between the amount of opioids administered intraoperatively and the probability for postoperative mechanical ventilation (*P* <0.05, Additional file 2: Table S2). The overall duration of surgery (*P* <0.001, Figure 3A) and the amount of blood loss (*P* <0.05, Figure 3B) also had a significant impact on the need for mechanical respiratory support (Additional file 2: Table S2). Comparing the postoperative course of patients with and without TEA, we found a significant difference in the amount of fentanyl given - patients with combined anaesthesia needed less fentanyl (*P* <0.001). We could not show any difference in the length of postoperative ventilation (*P* = 0.56), length of stay on the ICU (*P* = 0.52), nor time to first bowel passage (*P* = 0.73) between the two groups (Additional file 3: Table S3). However, the sample was strongly unbalanced, as sample sizes in the two groups were very different and data were missing. Regarding anaesthesia-related complications, one patient developed an epidural abscess after TEA, requiring operative decompression seven days after insertion.

### Major surgical complications (≥3b)

Major complications according to the Clavien-Dindo classification (grade 3b to 5) [22] occurred after 12 interventions, and 2 patients (4%) died. The first patient, a 46-year-old man, was suffering from adenocarcinoma of the esophagogastric junction and underwent transhiatal esophagogastrectomy. The effective operation time was 800 minutes. The patient required 8 units of packed red blood cells (PRBC), 500 IU prothrombin complex concentrate (PCC), and 10 g fibrinogen due to extensive bleeding (lowest hemoglobin level was 47 g l^−1^) intraoperatively. After an uneventful initial recovery, the patient died 17 days later from hemorrhagic shock and multiorgan dysfunction syndrome. The second patient, a 57-year-old man, was suffering from a mucinous adenocarcinoma of the appendix with peritoneal carcinomatosis. The effective operation time was 995 minutes. The surgery was complex and the patient required 4 units of PRBC (lowest hemoglobin level was 71 g l^−1^), 1 unit of platelets and several coagulation factor concentrates (14 g fibrinogen, 2500 IU factor XIII, 1000 IU PCC and 1000 IU factor VIII). After a long postoperative course with several re-interventions the patient died after 259 days from septic shock.

The rate of major surgical complications increased significantly with longer operation (Figure 3C) and anaesthesia time (both *P* <0.01; Figure 3D, Additional file 2: Table S2). We found the receipt of a blood transfusion to be an independent risk factor for major complications (*P* <0.05; Additional file 2: Table S2). The lowest overall hemoglobin values (describing the amount of blood loss) correlated with a trend towards an increased risk of major complications (*P* = 0.05, Additional file 2: Table S2). The administration of coagulation factor concentrates did not increase the risk of major complications, nor did the presence of obesity, arterial hypertension, carcinoma of the appendix, or preoperative anaemia (Additional file 2: Table S2).

## Discussion

Data on anaesthesia management and the outcome of 57 consecutive patients undergoing combined CRS/HIPEC were retrospectively collected and analyzed at our hospital. In addition to the individual surgical complexity, we have shown that several factors may affect patients’ outcome, such as the type and amount of resuscitation fluids used, as well as blood transfusions.

Cytoreductive surgery with HIPEC is a long-lasting, abdominal surgical procedure (median anaesthesia time 715 minutes) with additional hyperthermia and intraoperative chemotherapy. Extensive bleeding and fluid shifts may occur. Therefore, fluid status and cardiac function were continuously assessed with advanced hemodynamic monitoring in most of our patients.

Currently the type and amount of fluid administration is subject to debate [26-28]. Our fluid management consisted of both crystalloids and colloids. In addition to crystalloids, 51 patients received gelatine and 14 were also given HES, in a ratio of approximately 2.5:1. At the time of observation, studies on the potentially harmful effects of HES preparations in septic ICU patients had not yet been published [29,30]. Our data are in accordance with these publications: HES administration had a significant negative impact on renal function, especially in younger patients.

Maintaining renal function and prevention of injury is critical for obtaining the best perioperative outcome [31]. Known risk factors for acute renal injury are hypovolemia, hypotension, major surgery, nephrotoxic drugs, blood transfusions, and systemic inflammation [32]. Hemodynamic optimization (optimizing cardiac output, tissue perfusion, and oxygenation) is highly recommended to prevent renal injury. The goal is to maintain the effective circulating blood volume by careful fluid and transfusion management, vasopressors, and inotropes [33]. Most authors recommend liberal fluid regimens [14,18,34]. Our patients received approximately 10 ml kg^−1^ hr^−1^ of fluids and lost 3 ml kg^−1^ hr^−1^ (Additional file 3: Table 3). The amount of fluids given was guided by hemodynamic parameters, blood gas analyses, and urinary output. Most patients were given vasopressors to maintain MAP and, although the benefit of its application is questionable, 35 patients were given IV diuretics to force diuresis during HIPEC [33]. In fact, there is no evidence that a single pharmacological intervention during surgery protects the kidneys from damage [31,35].

Our data suggests that the need for a blood transfusion is associated with an increased risk for major complications (grade ≥3b according to the Clavien-Dindo classification [22]). The amount of bleeding showed a trend towards major complications (*P* = 0.05). It is standard procedure for both the surgical and the anaesthesia team to assess and estimate blood loss at the end of surgery. However, differences between both estimates result in inconsistent documentation. Alternatively, the decrease in hemoglobin concentration can be used as an indicator for blood loss. Both methods are widely used in clinics but are known to be of limited accuracy, tending to underestimate actual blood loss [36]. For future studies it might be useful to refer to a superior, validated blood loss score, taking into account the hemoglobin concentration of suction fluid [36].

Exposure to blood transfusions is associated with an increased morbidity and mortality in surgical oncology [37,38]. It is therefore critical to control surgical bleeding and to diagnose and correct coagulopathy early. Goal-directed, aggressive treatment using algorithms and point-of-care coagulation testing is recommended [37]. In our study, 28% of patients required intraoperative blood transfusions and 37% of patients were given coagulation factor concentrates. In contrast to other centers, routine FFP administration is not the first-line treatment for established coagulopathy at our institution [17]. Only 5% of patients received FFPs compared to 45% described in the literature [17,18]. The pathophysiology of coagulopathy in patients undergoing CRS/HIPEC is not completely understood [14,17]. Besides bleeding, consumption, and dilution, patients are exposed to extreme changes in body temperature (both hypo- and hyperthermia), suffer from metabolic acidosis, and calcium depletion (40% of our patients required calcium supplementation).

The use of TEA is recommended for patients undergoing CRS/HIPEC to provide optimal pain therapy, to reduce length of postoperative ventilation and pulmonary complications, and to allow for early mobilization (getting people moving as soon as possible) [14,34]. Critics underline the potential risk of hemodynamic instability, epidural hematoma, and infectious complications due to massive bleeding, impaired coagulation, and chemotherapy-induced immunodeficiency [39-41]. Recently an incidence ratio for infectious complications of 1:2139 has been reported [17]. One of our patients suffered from an epidural abscess with the need for an operative decompression seven days after placement. To prevent infections we recommend to limit the postoperative use of epidural analgesia to a maximum of five days and to visit patients with TEA daily. Despite the frequent use of TEA, only 28% of CRS/HIPEC centers describe their pain management as excellent [17]. Most of our patients received TEA for intra- and post-operative analgesia, and we found a significant opiod-sparing effect. However, unlike previous publications, we could not show that TEA was associated with a reduced length of postoperative ventilation and ICU stay, nor shortened time to first bowel passage [18].

The present observational study has some limitations. The anaesthesia management of patients did not follow strict protocols and there were no predefined exclusion criteria for the study. Furthermore, data were collected retrospectively and some data were missing due to absent documentation, compromising data analysis and reducing power of statistical conclusions.

## Conclusions

Taken together, combined CRS/HIPEC is a high-risk surgical procedure associated with major hemodynamic and metabolic changes. It requires coordinated and patient-centred anaesthetic management, including meticulous monitoring of the different physiological systems of the body. Besides primary disease and complexity of surgery, we have shown that the type and amount of fluids used, blood transfusions, and anaesthetic management may have an impact on patients’ outcome. To further differentiate factors affecting the outcome, prospective randomized controlled trials are highly warranted in this field.

## Abbreviations

BMI: body mass index; CRS: cytoreductive surgery; CVP: central venous pressure; FFP: fresh frozen plasma; GFR: glomerular filtration rate; HES: hydroxyl-ethyl starch; HIPEC: hyperthermic intraperitoneal chemotherapy; ICU: intensive care unit; MAP: mean arterial pressure; PACU: post-anaesthesia care unit; PCC: prothrombin complex concentrate; PRBC: packed red blood cells; SVV: stroke volume variation; TEA: thoracic epidural anaesthesia.

## Competing interests

The authors declare that they have no competing interests.

## Authors’ contributions

MTG initiated, planned and designed the study. MTG and MEK obtained ethic committee approval. MEK was responsible for data collection and analysis. RK and UH performed statistical analyses and revised the manuscript. KL was involved in data acquisition and patient recruitment. MEK wrote the first draft of the paper. MTG and BBS revised the manuscript. All authors have read and approved the final manuscript.

## Supplementary Material

Additional file 1: Table S1 Multiple linear regression models. Click here for file

Additional file 2: Table S2 Multiple logistic regression models. Click here for file

Additional file 3: Table S3 Wilcoxon rank sum tests. Click here for file
