# Supplementary material for: Anaesthesia in patients undergoing cytoreductive surgery with hyperthermic intraperitoneal chemotherapy: retrospective analysis of a single centre three-year experience
Source: World J Surg Oncol. 2014 May 1;12:136. doi: 10.1186/1477-7819-12-136 (PMC4113247; doi:10.1186/1477-7819-12-136)
Supplement: Additional file 1: Table S1 — Multiple linear regression models. [file 1477-7819-12-136-S1.docx]

*Table S 1*: Multiple linear regression models

| **Multiple linear regression model** | | | | |
| --- | --- | --- | --- | --- |
| **Dependent variable** | **Independent variable** | **Effect size** | **Standard error** | **p-value** |
| GFR postoperative day 1* | Preoperative GFR | 0.729 | 0.154 | **<0.001**^1^ |
|  | Age | -0.118 | 0.255 | 0.645 |
|  | BMI | -0.204 | 0.455 | 0.656 |
| GFR postoperative day 2* | Preoperative GFR | 0.809 | 0.155 | **<0.001**^1^ |
|  | Age | 0.198 | 0.257 | 0.445 |
|  | BMI | -0.184 | 0.459 | 0.69 |
| GFR postoperative day 1* | Intraoperative blood loss | -0.001 | 0.002 | 0.75 |
|  | Age | -0.131 | 0.26 | 0.618 |
|  | BMI | -0.202 | 0.459 | 0.663 |
|  | Preoperative GFR | 0.725 | 0.155 | **<0.001**^1^ |
| GFR postoperative day 2* | Intraoperative blood loss | <-0.001 | 0.002 | 0.884 |
|  | Age | 0.192 | 0.267 | 0.468 |
|  | BMI | -0.183 | 0.463 | 0.695 |
|  | Preoperative GFR | 0.807 | 0.157 | **<0.001**^1^ |
| GFR postoperative day 1* | Intraoperative urine secretion per time | -1.547 | 2.454 | 0.531 |
|  | Age | -0.128 | 0.257 | 0.620 |
|  | BMI | -0.214 | 0.458 | 0.643 |
|  | Preoperative GFR | 0.732 | 0.155 | **<0.001**^1^ |
| GFR postoperative day 2* | Intraoperative urine secretion per time | 1.254 | 2.477 | 0.615 |
|  | Age | 0.206 | 0.26 | 0.431 |
|  | BMI | -0.176 | 0.462 | 0.705 |
|  | Preoperative GFR | 0.806 | 0.156 | **<0.001**^1^ |
| GFR postoperative day 1* | Amount of cristalloids given over time (ml min^-1^) | -0.014 | 0.715 | 0.984 |
|  | Age | -0.118 | 0.258 | 0.649 |
|  | BMI | -0.204 | 0.46 | 0.659 |
|  | Preoperative GFR | 0.729 | 0.155 | **<0.001**^1^ |
| GFR postoperative day 2* | Amount of cristalloids given over time (ml min^-1^) | -0.768 | 0.713 | 0.286 |
|  | Age | 0.175 | 0.258 | 0.499 |
|  | BMI | -0.187 | 0.458 | 0.685 |
|  | Preoperative GFR | 0.813 | 0.155 | **<0.001**^1^ |
| GFR postoperative day 1* | Age*amount of HES given over time (ml min^-1^) | 0.949 | 0.204 | **<0.001**^1^ |
|  | Age | -0.56 | 0.236 | **0.022**^1^ |
|  | BMI | -0.411 | 0.39 | 0.297 |
|  | Preoperative GFR | 0.805 | 0.132 | **<0.001**^1^ |
|  | Amount of HES given over time | -52.54 | 11.124 | **<0.001**^1^ |
| GFR postoperative day 2* | Age*amount of HES given over time (ml min^-1^) | 0.95 | 0.204 | **<0.001**^1^ |
|  | Age | -0.245 | 0.236 | 0.305 |
|  | BMI | -0.375 | 0.39 | 0.341 |
|  | Preoperative GFR | 0.882 | 0.132 | **<0.001**^1^ |
|  | Amount of HES given over time | -53.906 | 11.116 | **<0.001**^1^ |
| Amount of transfused PRBC | Preoperative anemia (Hb<117g l^-1^) | -0.096 | 0.448 | 0.832 |
|  | Age | -0.001 | 0.015 | 0.956 |
|  | BMI | 0.012 | 0.031 | 0.7 |

GFR = Glomerular filtration rate, BMI = body mass index (kg m^-2^), HES = hydroxyethyl starch, PRBC = packed red blood cells

^1^Statistically significant

*box-cox-transformed
