# Supplementary material for: Anaesthesia in patients undergoing cytoreductive surgery with hyperthermic intraperitoneal chemotherapy: retrospective analysis of a single centre three-year experience
Source: World J Surg Oncol. 2014 May 1;12:136. doi: 10.1186/1477-7819-12-136 (PMC4113247; doi:10.1186/1477-7819-12-136)
Supplement: Additional file 2: Table S2 — Multiple logistic regression models. [file 1477-7819-12-136-S2.docx]

*Table S2:* Multiple logistic regression models

| **Multiple logistic regression model** | | | | |
| --- | --- | --- | --- | --- |
| **Dependent variable** | **Independent variable** | **Effect size** | **Standard error** | **p-value** |
| Postoperative ventilation | Amount of opiods (mg) | 0.919 | 0.391 | **0.018**^1^ |
|  | Age | 0.016 | 0.028 | 0.576 |
|  | BMI | -0.006 | 0.06 | 0.925 |
| Postoperatve ventilation | Overall duration of operation (min) | 0.014 | 0.004 | **<0.001**^1^ |
|  | Age | 0.043 | 0.043 | 0.316 |
|  | BMI | 0.015 | 0.076 | 0.84 |
| Postoperative ventilation | Blood loss (ml) | 0.002 | 0.001 | **0.003**^1^ |
|  | Age | 0.008 | 0.029 | 0.782 |
|  | BMI | 0.02 | 0.064 | 0.749 |
| Complications ≥ 3b | Operation time (min) | 0.006 | 0.002 | **0.004**^1^ |
|  | Age | -0.03 | 0.042 | 0.48 |
|  | BMI | -0.075 | 0.087 | 0.391 |
| Complications ≥ 3b | Anaesthesia time (min) | 0.006 | 0.002 | **0.005**^1^ |
|  | Age | -0.031 | 0.042 | 0.46 |
|  | BMI | -0.077 | 0.087 | 0.379 |
| Complications ≥ 3b | Lowest intraoperative haemoglobin value (g l^-1^) | -0.037 | 0.019 | 0.054 |
|  | Age | -0.048 | 0.034 | 0.157 |
|  | BMI | -0.052 | 0.08 | 0.515 |
| Complications ≥ 3b | Blood transfusion (0/1) | 1.76 | 0.724 | **0.015**^1^ |
|  | Age | -0.038 | 0.034 | 0.268 |
|  | BMI | -0.075 | 0.082 | 0.359 |
| Complications ≥ 3b | Coagulation factor (0/1) | 1.159 | 0.69 | 0.093 |
|  | Age | -0.044 | 0.034 | 0.192 |
|  | BMI | -0.066 | 0.081 | 0.414 |
| Complications ≥ 3b | Obesity (BMI>30 kg m^-2^) | 0.128 | 1.6 | 0.936 |
|  | Age | -0.042 | 0.032 | 0.188 |
|  | BMI | -0.072 | 0.106 | 0.501 |
| Complications ≥ 3b | Hypertension | 0.778 | 1.082 | 0.472 |
|  | Age | -0.049 | 0.034 | 0.148 |
|  | BMI | -0.094 | 0.089 | 0.29 |
| Complications ≥ 3b | Carcinoma of the appendix | 0.1 | 0.685 | 0.886 |
|  | Age | -0.042 | 0.032 | 0.186 |
|  | BMI | -0.067 | 0.077 | 0.38 |
| Complications ≥ 3b | Pre-operative anaemia  (Hb<117g l^-1^) | 1.19 | 0.872 | 0.173 |
|  | Age | -0.048 | 0.032 | 0.129 |
|  | BMI | -0.08 | 0.078 | 0.313 |

^1^Statistically significant

BMI = body mass index (kg m^-2^), Hb = haemoglobin concentration (g l^-1^)
