# Supplementary material for: Anaesthesia in patients undergoing cytoreductive surgery with hyperthermic intraperitoneal chemotherapy: retrospective analysis of a single centre three-year experience
Source: World J Surg Oncol. 2014 May 1;12:136. doi: 10.1186/1477-7819-12-136 (PMC4113247; doi:10.1186/1477-7819-12-136)
Supplement: Additional file 3: Table S3 — Wilcoxon rank sum tests. [file 1477-7819-12-136-S3.docx]

*Table S 3*: Wilcoxon rank sum tests

| **Wilcoxon rank sum tests** | | | | |
| --- | --- | --- | --- | --- |
| **Dependent variable** | **Groups** | **N** | **Mean** | **p-value** |
| Fentanyl given intraoperatively (mg)  n=57 | Additional TEA versus no additional TEA | n=45  versus  n=12 | 0.015 versus 0.03471854 | <0.001^1^ |
| Length of postoperative ventilation (h)  in n=27** patients | Additional TEA versus no additional TEA | n=20  versus n=7 | 5.013  versus  4.536 hours | 0.560 |
| Total stay on ICU (days) in n=53 patients | Additional TEA versus no additional TEA | n=42  versus n=11 | 3.024  versus 1.545 days | 0.519 |
| Time to first bowel passage (days)  in n=55 patients*** | Additional TEA versus no additional TEA | N=44  versus n=11 | 5.3409 versus 5.7272 days | 0.732 |

^1^Statistically significant

**data missing in n=6 patients

*** data missing in n=2 patients

TEA = thoracic epidural anaesthesia, ICU = intensive care unit
